# Supplementary material for: In situ dynamic tracking of heterogeneous nanocatalytic processes by shell-isolated nanoparticle-enhanced Raman spectroscopy
Source: Nat Commun. 2017 May 24;8:15447. doi: 10.1038/ncomms15447 (PMC5458081; doi:10.1038/ncomms15447)
Supplement: Supplementary Information — Supplementary Figures, Supplementary Tables, Supplementary Methods and Supplementary References. [file ncomms15447-s1.pdf]

Supplementary Methods

Synthesis of 120 nm Au cores. 120 nm Au cores were synthesized by a seed-mediated growth method. First, 45 nm Au nanoparticles were prepared according to Frens' method[1] and used as seeds. 3 mL of the as-prepared dispersion of 45 nm Au nanoparticles, 0.4 mL of 1 wt% ascorbic acid and 0.1 mL of 1 wt% sodium citrate were then added to 20 mL of ultrapure water and stirred in an ice bath. 3.3 mL of 1 wt% chloroauric acid were added to this solution dropwise over a period of 30 min. The resulting dispersion was stirred vigorously at 70 ^oC for 20 min to form very uniform, spherical Au nanoparticles with diameters around 120 nm.

Synthesis of nanocatalysts. Pt, Pd[2], PtPd[3], PtFe[4], Au@PtFe[5], CeO_2[6] and Fe_2O_3[7] were prepared by following published procedures.

Pt, Pd, and PtPd. Pt was synthesized using borane *tert*-butylamine complex (BTB) as reducing agent, and oleylamine as both solvent and capping agent. Typically, 50 mg platinum (II) acetylacetonate ($Pt(acac)_2$) were dissolved in 15 mL oleylamine. After heating 120 ^oC, 200 mg BTB in 2 mL oleylamine were injected. Then, the reaction temperature was raised to 140 ^oC, and kept for 1 h. After cooling down to room temperature, the mixtures were washed with ethanol for several times. The obtained solid was Pt nanocatalysts, which was then dispersed in toluene or hexane for further experiments. Pd and PtPd were synthesized via the similar procedure for Pt.

PtFe, PdFeCu nanocube. PtFe were prepared through a thermal decomposition method. $Pt(acac)_{2,}$ iron(III) acetylacetonaten ($Fe(acac)_3$), oleylamine, and oleic acid were dissolved in benzyl ether. The mixtures were heated to 270 ^oC under Ar, and kept at this temperature for 1.5 h. The obtained products were then washed with ethanol, and dispersed in toluene or hexane. PdFeCu nanocubes were synthesized using the similar procedure, but with palladium(II) acetylacetonatean ($Pd(acac)_2$), copper(II) acetylacetonate ($Cu(acac)_2$), and Fe-oleate complex[8] as precursors.

Au@PtFe. Au@PtFe was prepared via a seed-mediated method. Au nanoparticles were first prepared in oleylamine at room temperature, using BTB as reducing agent. The obtained Au nanoparticles were then dispersed in benzyl ether, along with $Pt(acac)_{2,}$ $Fe(acac)_3$, oleylamine, and oleic acid. The mixtures were heated to 270 ^oC under Ar, and kept at this temperature for 1.5 h to generate Au@PtFe nanocatalysts. The obtained products were then washed with ethanol, and dispersed in toluene or hexane.

CeO_2. Cerium (III) nitrate, oleic acid, *tert*-butylamine, and toluene were added to a autoclave. The autoclave was sealed and heated to 180 ^{o}C, and kept at this temperature for 24 h. After cooling to room temperature, the mixtures were centrifuged to remove the solid impurities. CeO_2 nanoparticles were then obtained by precipitation of the supernatant solution with excess ethanol. The obtained CeO_2 nanoparticles were then washed with ethanol for several times, and dispersed in toluene or hexane.

Fe_2O_3. $Fe(acac)_3$ was dissolved in oleylamine, and heated to 300 ^{o}C under Ar. After reacting for 2 h at this temperature, the mixtures were cooled down to room temperature. The products containing Fe_2O_3 nanoparticles were then washed with ethanol for several times. The purified Fe_2O_3 nanoparticles were dispersed in toluene or hexane.

3D-FDTD simulations. These calculations are based on the discretization of Maxwell's difference equations. Space and time are divided into discrete segments. Space is segmented into box-shaped cells, with electric fields located on the edges and magnetic fields located on the faces so that the electric and magnetic fields can be obtained at different positions in time. This orientation of the fields is known as a Yee cell. A standard Cartesian Yee cell used in 3D-FDTD calculations is often set as a cubic voxel, and the 3D space lattice is comprised of a multiplicity of such Yee cells. In order to obtain an accurate field distribution for a 3D object, the Yee cell size ($\Delta s\times\Delta s\times\Delta s$, where Δs is the side-length of each cell), which is the most important constraint in any 3D-FDTD simulation, must be much less than the excitation wavelength (λ).

$$\Delta s \leq \lambda/12 \qquad (1)$$

In this work, the simulations were conducted using the commercially available Lumerical Solutions software (version 7.5). Two close-packed SHINERS-satellite nanocomposites with 120 nm Au cores, 2 nm silica shells and 2 nm Pt nanocatalysts were modelled. In order to ensure calculation convergence and accuracy, the simulation time and Yee cell size were set at 1000 fs and 0.5 nm respectively.

DFT calculation method. Spin-unpolarized calculations were carried out at the level of RPBE[9] using the Vienna ab initio simulation package (VASP 5.3.5)[10,11]. It has been

well documented that the commonly used density functionals, such as LDA, PBE, PW91 and HSE, overestimated the adsorption energies of CO on metal surface[12-14]. RPBE functional was specially designed to remedy the notorious problem. Gajdos et al.[13] found that for the late transition metal surface, the predicted adsorption energies by using RPBE agreed well with the experimental values. Additionally, Schimka et al.[14] pointed out that RPBE had similar accuracy to newly developed random phase approximation (RPA) for CO adsorption on top site of Pt(111) and Rh(111). Our test calculations showed that CO is preferentially adsorbed on the hollow site of Pd(111) with the adsorption energy of -1.67 eV at 1/4 monolayer (ML) coverage, in good agreement with previous theoretical prediction (-1.68 eV) and experimental value (-1.42 eV). It should be noted that the adsorption energy for CO/Pd(111) listed in Supplementary Table 2 was at 1/16 ML coverage. The valence electrons were described by plane wave basis sets with a cut-off energy of 400 eV, and the core electrons were replaced by the projector augmented wave pseudopotentials[15,16].

For a clean Pd(111) surface, a (4×4) supercell with five layer slabs was used (Supplementary Fig. 22a). During structural optimization, the bottom two layer slabs were fixed at a bulk truncated position, while the top three layer slabs and the adsorbates were fully relaxed. The exposure of Pd(111) to oxygen above room temperature would result in different kinds of surface oxides, such as Pd(111)-O, Pd_5O_4 and PdO(100)-O[17]. Here, Pd(111)-O was modelled as a well-ordered p(2×2) oxygen adlayer (a quarter monolayer of O atoms) on Pd(111) (Supplementary Fig. 22b). Pd_5O_4 was modelled as a two-dimensional oxide film on a hexagonal Pd(111) substrate, which had a square unit cell with side lengths equal to $\sqrt{6}$ times that of the Pd-Pd distance[18]. Pd_5O_4 formed a large unit cell and had a surface oxygen coverage of 0.67 monolayers (Supplementary Fig. 22c). For the sake of computational efficiency, two layers of Pd were used to model the Pd(111) substrate with the bottom layer fixed. PdO(100)-O was modelled as a PdO(100) surface with excess oxygen (Supplementary Fig. 22d)[19], which is considered to have a rather low surface energy. In this case, every Pd atom was coordinated with two oxygen atoms, resulting in 2 monolayers of oxygen coverage. Here we used a (2×2) supercell with five layer slabs to model the PdO(100)-O surface and kept the bottom two layers fixed. For all of the calculations, the vacuum regions between the slabs were more than 10 Å, and Monkhurst-Pack k-point sampling with approximately $0.05 \times 2\pi$ Å^{-1} spacing in a reciprocal lattice was utilized.

The minimum energy reaction pathways were calculated using the nudged elastic band method. The final transition state structures were refined using a quasi-Newton algorithm until the Hellman-Feynman forces on each ion were lower than 0.03 eV/Å. The adsorption energies (ΔE_{ads}) were calculated using equation 2, in which $E_{ad/sub}$, E_{ad}, and E_{sub} were the total energies of the optimized adsorbate/substrate system, the adsorbate in the gas phase, and the clean substrate respectively.

$$\Delta E_{ads} = E_{ad/sub} - E_{ad} - E_{sub} \quad (2)$$

It is known that triplet O_2 is poorly described by GGA functionals. Similar to previous work[20], we used gas-phase H_2O and H_2 as references to estimate the total energy of O_2 with equation 3. In equation 3, ΔE_r denoted the reaction heat at 0 K without a zero-point energy correction, which can be deduced from the experimental atomic energies[9] of O_2, H_2 and H_2O using equation 4. In equation 4, $AE(H_2O)$, $AE(H_2)$ and $AE(O_2)$ represented the atomic energies of H_2O, H_2 and O_2, respectively. Our calculations showed that the corrected total energy of O_2 was -9.91 eV.

$$E(O_2) = 2\times E(H_2O)-2\times E(H_2)-\Delta E_r \quad (3)$$

$$\Delta E_r = 2\times AE(H_2O)-2\times AE(H_2)-AE(O_2) \quad (4)$$

Supplementary Figures

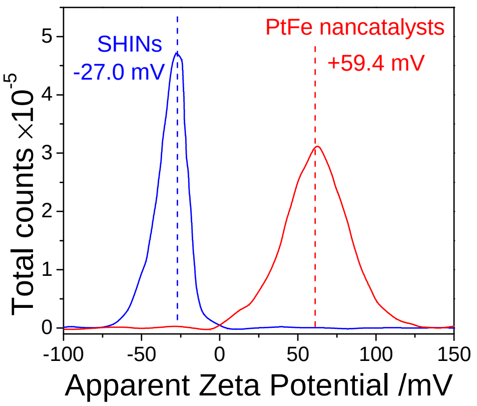

Supplementary Figure 1|Zeta potentials for the as-synthesized SHINs and the modified PtFe nanocatalysts.

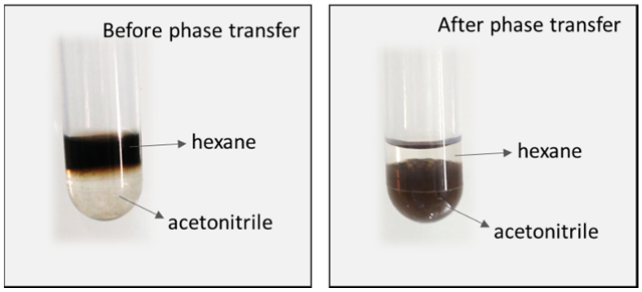

Supplementary Figure 2|Photographs of the dispersions of the PtFe nanoalloy before and after phase transfer. This phase-transfer process can be observed as the dark brown colour moves from the hexane layer to the acetonitrile layer.

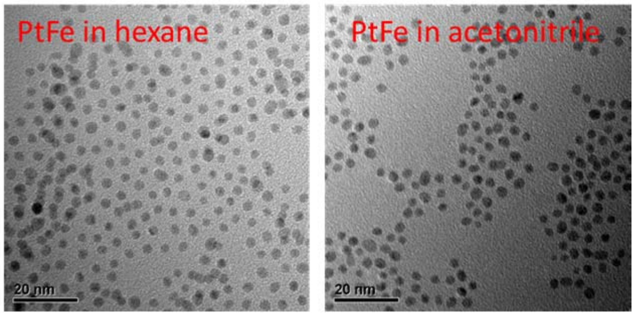

Supplementary Figure 3|TEM images of the PtFe nanoalloy before and after phase transfer. No aggregation occurred after the phase transfer, and the size and morphology of the PtFe nanocatalysts remained unchanged. Scale bars, 20 nm.

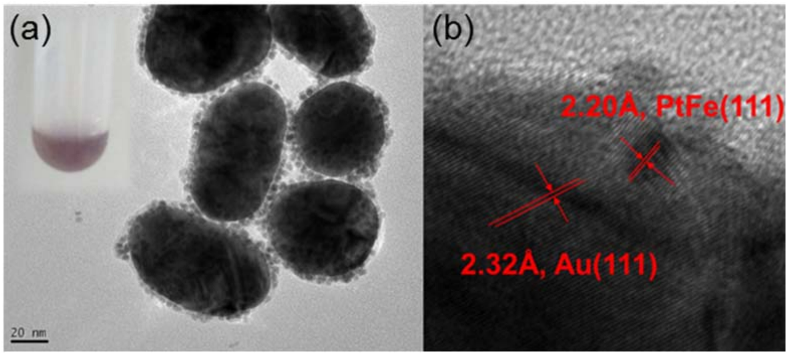

Supplementary Figure 4 | Typical TEM (a) and HR-TEM (b) images of PtFe-on-SHIN nanostructures. The inset shows a photograph of a solution of PtFe-on-SHINs in acetonitrile, which can remain stable for several weeks without any precipitation. Scale bar, 20 nm.

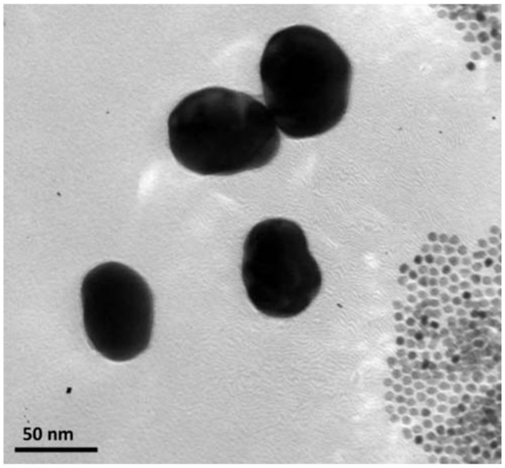

Supplementary Figure 5 | TEM image of a mixture of PtFe nanoparticles and SHINs. The mixture was obtained by adding a solution of PtFe nanoparticles in toluene (they were not modified with $NOBF_4$ and were still protected by oleylamine and oleic acid) to a solution of SHINs in acetonitrile. Phase separation between the PtFe nanoparticles and the SHINs was clearly observed. This is reasonable because the surface of the PtFe nanoparticles was still protected by oleylamine and oleic acid, and therefore electrically neutral. This finding indicates that modification of PtFe nanoparticles to create a positively charged surface is critical for the synthesis of SHINERS-satellite nanocomposites.

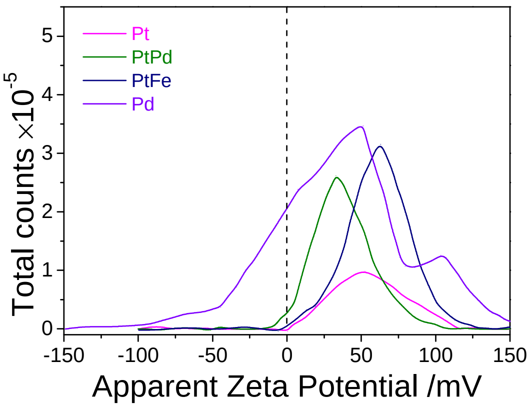

Supplementary Figure 6 | Zeta potentials for various nanocatalysts after surface modification with $NOBF_4$. All of the catalysts have a positively charged surface after modification, indicating that charged-induced self-assembly can be a general method for the construction of SHINERS-satellite nanocomposites.

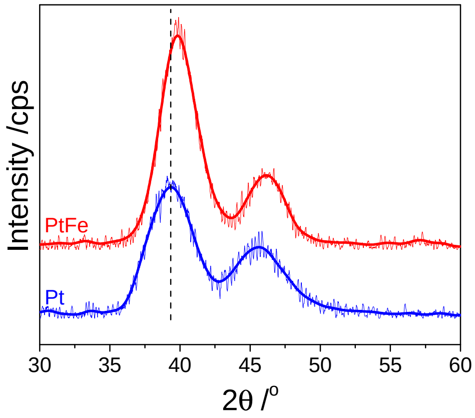

Supplementary Figure 7 | XRD patterns obtained from Pt and PtFe nanocatalysts. The diffraction peaks for the PtFe bimetallic catalyst are shifted to higher values, indicating that Fe forms an alloy with Pt.

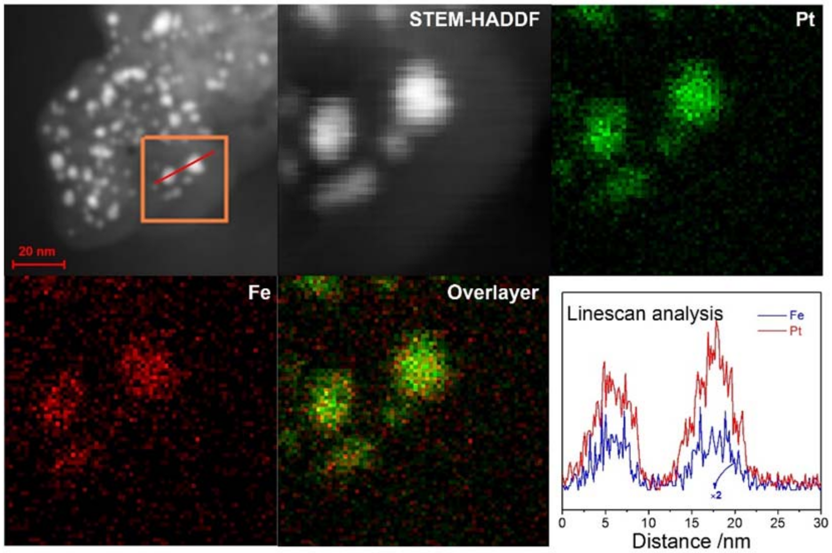

**Supplementary Figure 8 | ** Element maps and line scans obtained from the PtFe nanoalloy.

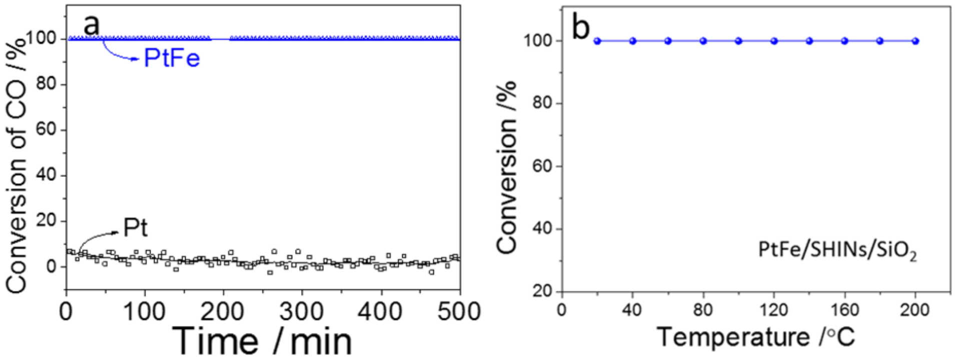

Supplementary Figure 9 | a, Catalytic performance results showing preferential oxidation of CO over PtFe/SiO_2 and Pt/SiO_2 at 30 ^{o}C in the presence of H_2 (1% CO, 1% O_2, 50% H_2, balance N_2; weight hourly space velocity of 80,000 mL·g^{-1}·h^{-1}). **b,** Catalytic performance results for CO oxidation over PtFe/SHINs/SiO_2 (1% CO, 21% O_2, balance N_2; weight hourly space velocity of 80,000 mL·g^{-1}·h^{-1}). It can be seen that the addition of SHINs does not change the performance, as CO is still completely converted to CO_2, even at room temperature.

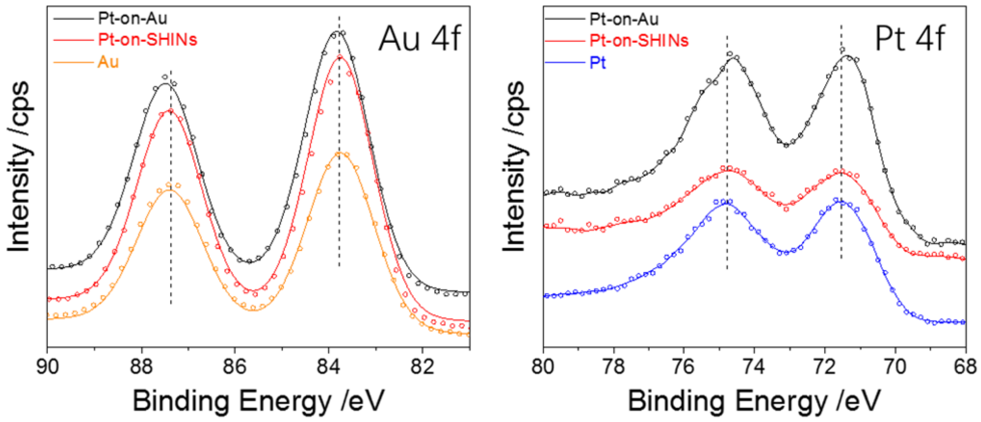

Supplementary Figure 10 | XPS spectra of Pt nanocatalysts assembled on Au nanoparticles and SHINs. The binding energies of Au 4f and Pt 4f for the Pt-on-SHIN structures are almost the same as those for the component Au nanoparticles and Pt nanocatalysts, respectively. This means that the electronic interactions between Pt and Au for Pt-on-SHINs are negligible. However, the binding energies of Au 4f shift to higher values while those of Pt 4f shift to lower values compared to pure Au and Pt, respectively, if Pt nanocatalysts are assembled directly on bare Au nanoparticles (Pt-on-Au). This indicates that electrons are transfered from Au to Pt in the Pt-on-Au structure. From these results, we conclude that the silica shells prevent electronic interactions between the Au cores and the catalysts, and that the intrinsic behavior of the catalysts is preserved.

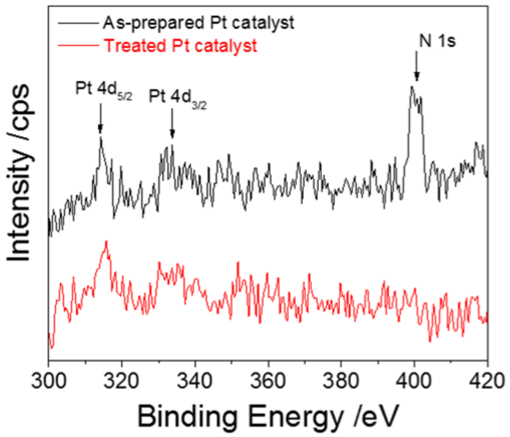

Supplementary Figure 11 | XPS spectra of the as-prepared Pt nanocatalysts, as well as the Pt-on-SHIN structures before *in-situ* SHINERS-satellite studies. Pt nanocatalysts are synthesized using oleylamine as a capping agent, which is then replaced by $NOBF_4$ during the surface modification process. $NOBF_4$ binds to the surface much more weakly, and can easily be replaced or removed[21]. Furthermore, the catalyst-on-SHIN structures are treated with H_2 before the *in-situ* SHINERS studies, and this will further clean the catalyst surfaces. An XPS signal is observed for N 1s on the as-prepared Pt catalysts, which is from the NH_2- group of the adsorbed oleylamine. This signal disappears from the treated catalyst, indicating that the oleylamine molecules are removed from the catalyst surface.

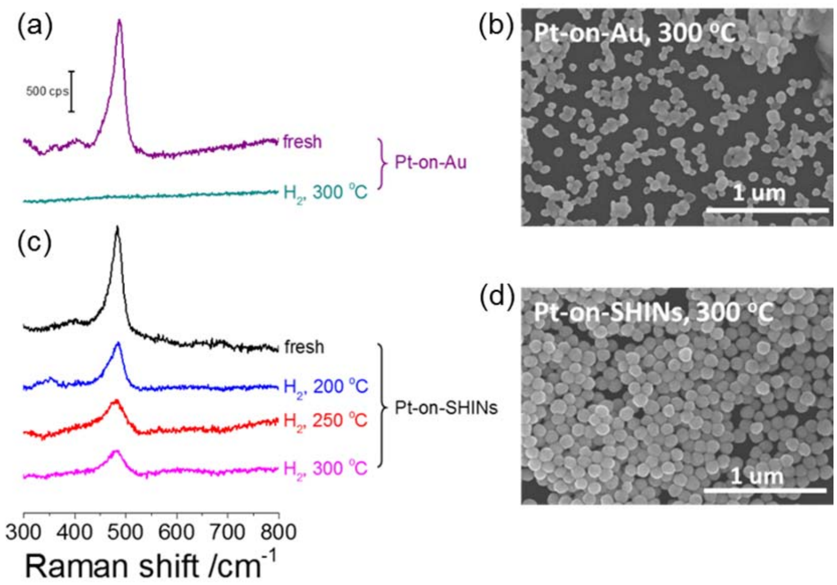

Supplementary Figure 12 | Raman spectra of CO adsorbed on Pt-on-Au and Pt-on-SHIN nanostructures at elevated temperatures (a, c); and SEM images of Pt-on-Au and Pt-on-SHIN nanostructures after they were heated to 300 ^{o}C for 30 min (b, d). The Pt-on-Au structures were heated at a temperature of 300 ^{o}C under H_2 for 30 min, cooled to room temperature, then placed under CO for the acquisition of Raman spectra. No bands were seen. When this procedure was repeated for Pt-on-SHIN structures, a Pt-C stretching band from CO on Pt was observed. The SEM images show that the Pt-on-Au structures coalesce at high temperature, but the silica shell prevents the Pt-on-SHIN structures from doing so. When this process was repeated for Pt-on-SHIN structures at a couple of additional temperatures, the intensity of the Pt-C stretching band was found to decrease with increasing temperature. These results show that although the Pt-on-SHIN structure is somewhat sensitive to temperature, it is far more stable than the Pt-on-Au structure. These results are important for applications in heterogeneous catalysis, which usually operate at high temperatures.

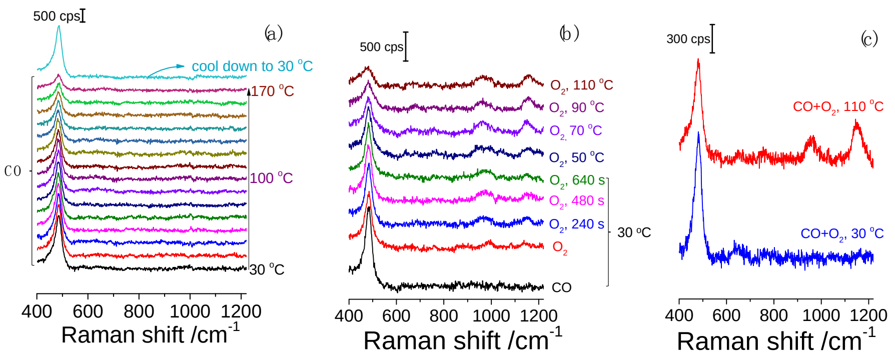

Supplementary Figure 13 | Raman spectra of CO and O_2 on Pt-on-SHIN nanostructures as a function of temperature: (**a**) adsorption of CO at increasing temperatures, (**b**) adsorption of CO followed by a change in the feed to pure O_2 and then an increase in temperature, (**c**) CO oxidation at 30 and 110 ^oC. The intensity of the Pt-C stretching band decreases rapidly as temperature increases, and no additional bands appear (Supplementary Fig. 13a). This indicates that CO desorbs from the catalyst surface and CO coverage decreases as temperature increases. If the feed is changed to pure O_2 after initial adsorption of CO, Raman bands for oxygen species appear (Supplementary Fig. 13b). This result means that oxygen easily adsorbs on the catalyst surface if pure O_2 is present in the feed. With prolonged reaction times and increased temperatures, the intensities of the oxygen species peaks increase while the intensity of the Pt-C peak decreases (Supplementary Fig. 13b). *In-situ* SHINERS-satellite spectra of CO oxidation on Pt nanocatalysts at 30 ^oC show only the Pt-C stretching band (Fig. 3c blue curve and Supplementary Fig. 13c). From this we conclude that the activation of O_2 is inhibited by CO, as explained by the DFT results of Bao et al.[22] They found that the adsorption energy of CO on Pt (-1.64 eV) is much lower than that of O_2 on Pt (-0.71 eV). Thus, CO tends to cover the Pt surface and block O_2 adsorption. At elevated temperatures, CO will desorb from the Pt surface and allow O_2 adsorption on some of the active sites. Thus, Raman signals for both CO and O_2 are seen during CO oxidation at 110 ^oC (Supplementary Fig. 13c), and the activity increases.

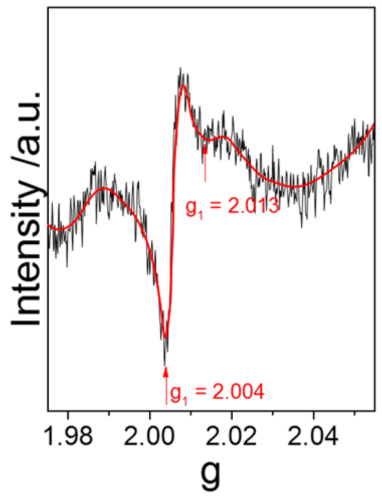

Supplementary Figure 14 | An EPR spectrum obtained from PtFe nanoalloy catalysts after CO oxidation. The catalysts were removed from the fixed-bed lab reactor, immediately sealed in an NMR tube with a gas mixture that was similar in composition to the one used for CO oxidation, then placed in an EPR spectrometer as quickly as possible. The two narrow peaks at about 2.004 and 2.013 can be assigned to the O_2^- species[23,24]. Fe^{3+} species, which show a very broad peak at about g = 2.0, are not observed. These results are consistent with the XPS results.

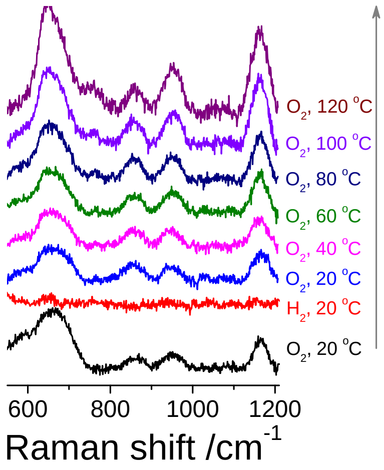

Supplementary Figure 15 | Spectra from SHINERS-satellite structures with Pd nanocatalysts under an O_2 or H_2 atmosphere. Three peaks were present at about 860, 955 and 1165 cm^{-1} when O_2 adsorbed on the Pd nanocatalysts. They disappeared immediately when the feed was changed to H_2, and appeared again when the feed was returned to O_2. These results indicate that the three peaks are from species closely related to oxygen. We note that their Raman intensities increased with increasing temperature.

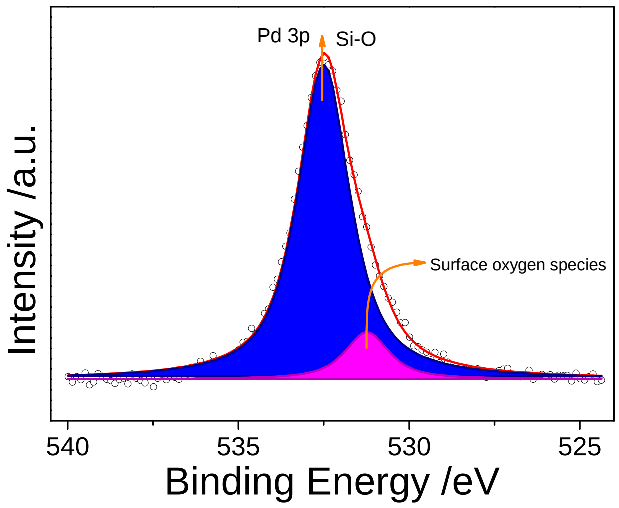

Supplementary Figure 16 | An XPS study of SHINERS-satellite structures with Pd nanocatalysts after exposure to O_2 at 150 ^{o}C. According to the literature[25,26], the peak

at 531.2 eV can be assigned to O 1s of surface oxygen species, and the peak at 532.5 eV can be assigned to Pd 3p of the Pd nanocatalysts and O 1s of Si-O-Si.

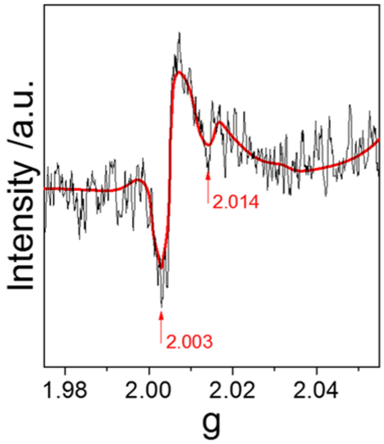

Supplementary Figure 17| An EPR spectrum obtained from a Pd catalyst after exposure to O_2. It shows two peaks at around 2.003 and 2.014, which can be assigned to surface O_2^- species[23,24].

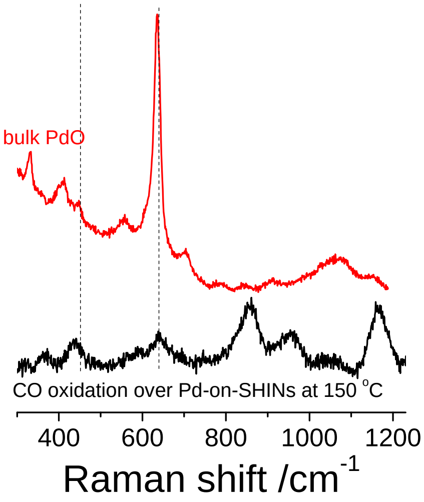

Supplementary Figure 18| A normal Raman spectrum obtained from bulk PdO powder at 25 °C (red curve), and a SHINERS-satellite spectrum obtained for CO oxidation on Pd nanocatalysts at 150 °C (black curve).

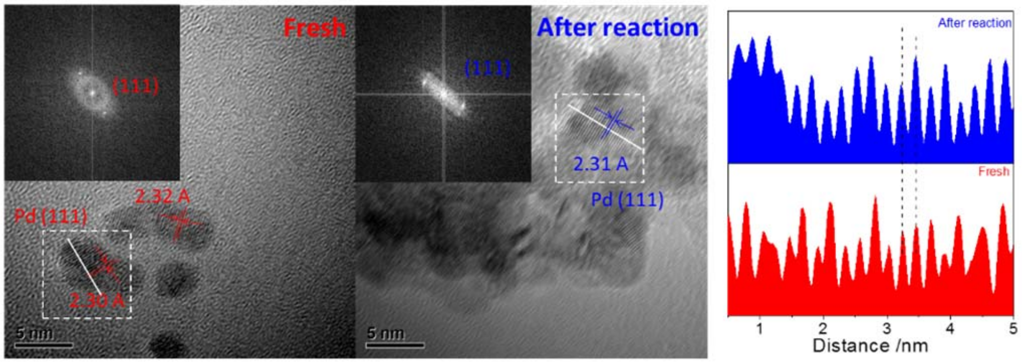

Supplementary Figure 19|HR-TEM images of Pd nanocatalysts before and after CO oxidation at 150 °C. Scale bars, 5 nm.

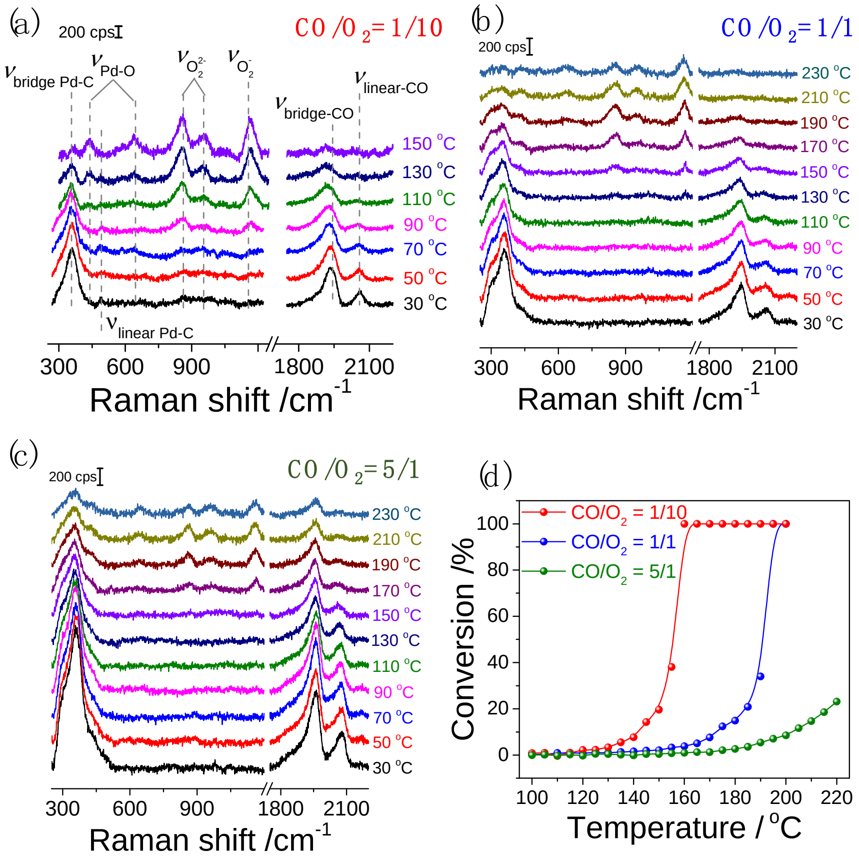

Supplementary Figure 20|SHINERS-satellite spectra for CO oxidation over Pd nanocatalysts with different gas ratios in the feed: (**a**) CO/O_2=1/10, (**b**) CO/O_2=1/1, (**c**) CO/O_2=5/1. Catalytic performance for CO oxidation over Pd nanocatalysts under different feed conditions (**d**).

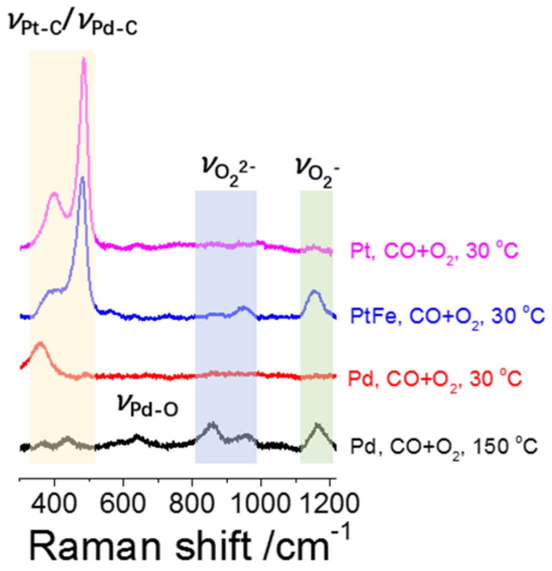

Supplementary Figure 21 | SHINERS-satellite spectra obtained for CO oxidation over Pt, Pd and PtFe nanocatalysts under oxygen rich conditions. CO is mainly adsorbed on Pt in a linear configuration and on Pd in a bridged configuration. This is because the adsorption energy for linear CO on Pt is more negative than that for bridged CO on Pt, while the opposite is true for Pd[27]. Raman bands for Pt-C and Pd-C stretching modes are observed for CO on Pt and Pd respectively at 30 °C, and no signals are observed for oxygen species. Almost all of the Pt and Pd catalyst surfaces are covered by CO at this temperature, and the sites for O_2 activation are blocked. For the PtFe bimetallic catalyst, the adsorption energies for CO and O_2 are comparable[22]. Therefore, CO and O_2 are co-adsorbed on the catalyst and the reaction can proceed at room temperature by the Langmuir-Hinshelwood mechanism. As for CO oxidation on Pd nanocatalysts at higher temperatures, CO begins to desorb and the Pd surface becomes oxidized to PdO_x. This leads to the availability of active sites on which O_2 can adsorb. Thus, CO oxidation can proceed on Pd at high temperatures by the Eley-Rideal mechanism.

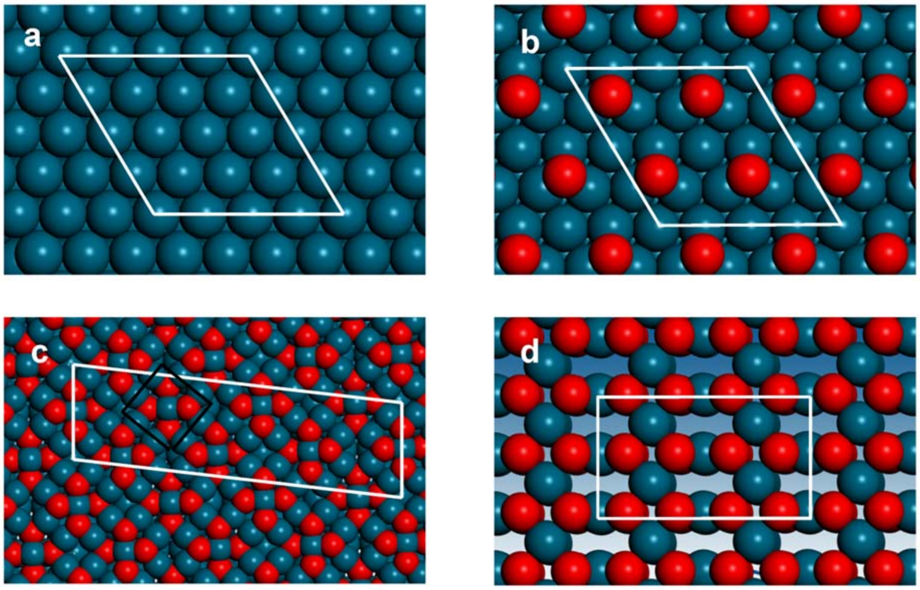

Supplementary Figure 22|Theoretical models of metallic Pd and surface oxides. **a,** a Pd(111) surface; **b,** a Pd(111)-O surface with a p(2×2) oxygen adlayer; **c,** a two dimensional Pd_5O_4 surface oxide; **d,** a PdO(100)-O surface with excess oxygen atoms.

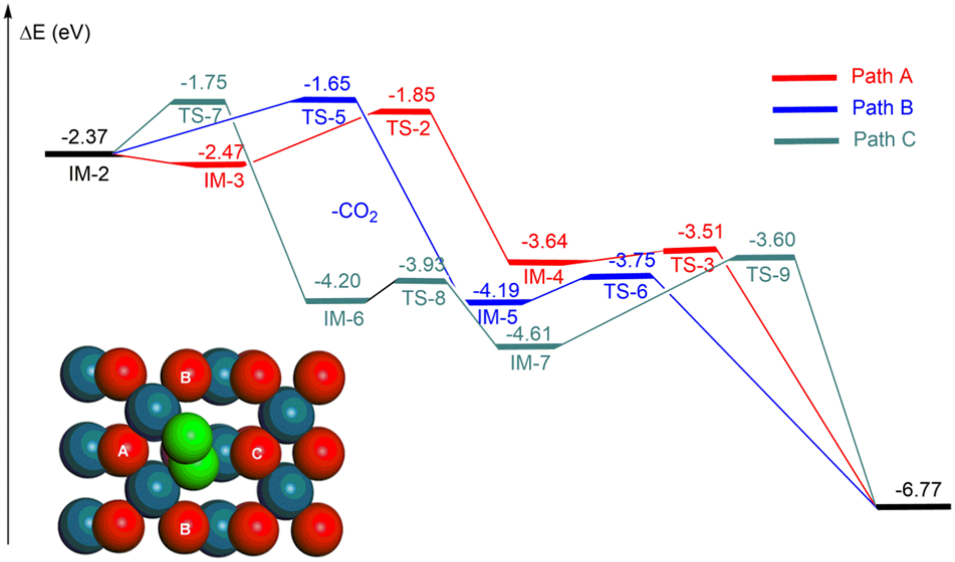

Supplementary Figure 23|Possible mechanisms for the second CO oxidation and the O-O bond dissociation (inset: A, B and C are three possible oxygen sites near the adsorbed O_2).

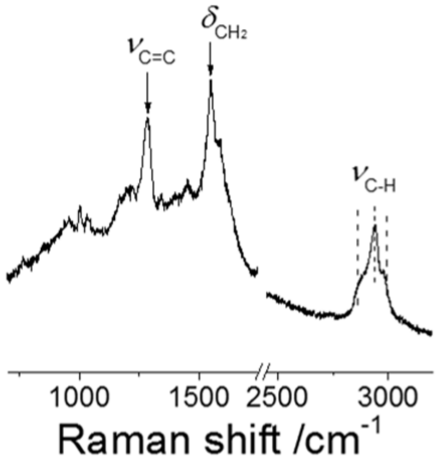

Supplementary Figure 24 | SHINERS-satellite spectrum of ethylene adsorbed on Pd nanocatalysts. The Raman bands at 1280, 1550, and 2840-3040 cm^{-1} can be attributed to the coupled C=C stretch, CH_2 scissor, and C-H stretch modes of ethylene on Pd[28].

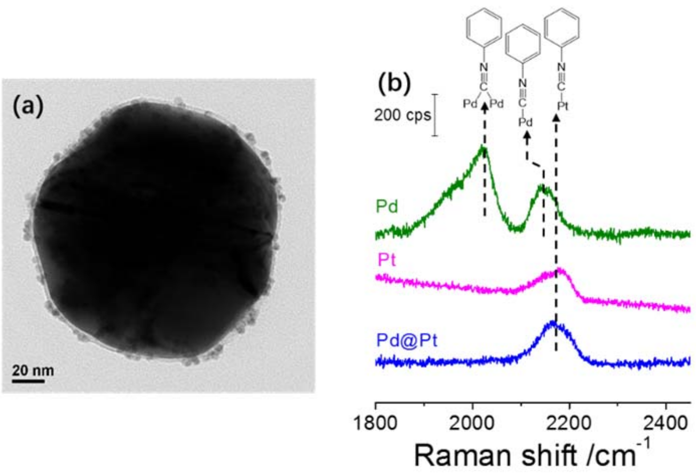

Supplementary Figure 25 | (a) A TEM image showing a Au-core silica-shell Pd@Pt-nanocatalyst structure. (**b**) SHINERS-satellite spectra of phenyl isocyanide adsorbed on Pd, Pt and Pd@Pt nanocatalysts. The composition of the outermost atomic layers of the Pd@Pt core-shell catalyst can be determined using phenyl isocyanide as a probe molecule. Two Raman bands at about 2020 and 2150 cm^{-1} are observed for the Pd nanocatalyst, which can be assigned to the C≡N stretching modes of bridge and linearly adsorbed phenyl isocyanide respectively.[29] Only one Raman band at about 2170 cm^{-1} is observed for the Pt nanocatalyst, which can be assigned to the C≡N stretching mode of linearly adsorbed phenyl isocyanide. The distinct differences in the Raman spectra of phenyl isocyanide on Pd and Pt allow the surface composition of the Pd@Pt core-shell catalyst to be elucidated. The single Raman band at about 2170 cm^{-1} in the SHINERS-satellite spectrum of phenyl isocyanide on Pd@Pt indicates that, as expected, only Pt atoms are present on the surface.

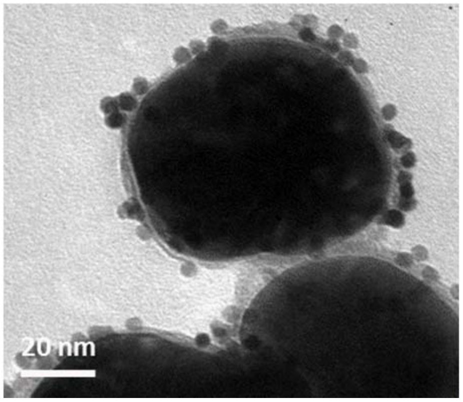

Supplementary Figure 26 | A TEM image showing Ag-core silica-shell Pt-nanocatalyst structures.

Supplementary Tables

Supplementary Table 1 | Calculated adsorption energies, O-O bond distances, charges, vibrational frequencies and spin densities for O_2 on different surfaces and at different sites

Surface[a]	Site[b]	Adsorption energy (eV)	D_{O-O} (Å)	Charge (a.u.)	Frequency[c] (cm^{-1})	O_2 spin density
Pd(111)	hollow	-1.26	1.369	-0.56	946	0.01
Pd(111)-O	hollow	-0.48	1.338	-0.48	948	0.31
Pd_5O_4	bridge	-0.64	1.284	-0.32	1163	1.04
Pd_5O_4-vac	open	-1.54	1.402	-0.72	806	0.00
	close	-1.51	1.397	-0.73	864	0.00
PdO(100)-O-vac	open	-0.84	1.381	-0.64	875	0.05
	close	-0.81	1.354	-0.58	944	0.38

[a] The suffix "vac" indicates that there is an oxygen vacancy on the surface.

[b] "Open" and "close" indicate the adsorbed O_2 molecules would open or close vacancies on the surface oxides.

[c] The frequencies have been scaled by a factor of 0.95.

Supplementary Table 2 | Calculated adsorption energies and C-O bond distances for CO on different surfaces and at different sites

Surface[a]	Site	Adsorption energy (eV)	D_{C-O} (Å)
Pd(111)	hollow	-2.05	1.198
Pd(111)-O	hollow	-1.99	1.198
Pd_5O_4	bridge	-0.76	1.175
Pd_5O_4-vac	bridge	-1.67	1.185
PdO(100)-O-vac	hollow	-0.81	1.180

[a] The suffix "vac" indicates that there is an oxygen vacancy on the surface.

Supplementary References

1. Frens, G. Controlled nucleation for the regulation of the particle size in monodisperse gold suspensions. *Nature* **241**, 20-22 (1973).
2. Mazumder, V. & Sun, S. Oleylamine-mediated synthesis of Pd nanoparticles for catalytic formic acid oxidation. *J. Am. Chem. Soc.* **131**, 4588-4589 (2009).
3. Liu, Y. *et al.* Composition-controlled synthesis of bimetallic PdPt nanoparticles and their electro-oxidation of methanol. *Chem. Mater.* **23**, 4199-4203 (2011).
4. Nandwana, V. *et al.* Size and shape control of monodisperse FePt nanoparticles. *J. Phys. Chem. C* **111**, 4185-4189 (2007).
5. Sun, X. *et al.* Core/shell Au/CuPt nanoparticles and their dual electrocatalysis for both reduction and oxidation reactions. *J. Am. Chem. Soc.* **136**, 5745-5749 (2014).
6. Yang, S. & Gao, L. Controlled synthesis and self-assembly of CeO_2 nanocubes. *J. Am. Chem. Soc.* **128**, 9330-9331 (2006).
7. Yu, Y. *et al.* Monodisperse MPt (M = Fe, Co, Ni, Cu, Zn) nanoparticles prepared from a facile oleylamine reduction of metal salts. *Nano Lett.* **14**, 2778-2782 (2014).
8. Park, J. *et al.* Ultra-large-scale syntheses of monodisperse nanocrystals. *Nat. Mater.* **3**, 891-895 (2004).
9. Perdew, J. P., Burke, K. & Ernzerhof, M. Generalized gradient approximation made simple. *Phys. Rev. Lett.* **77**, 3865-3868 (1996).
10. Kresse, G. & Hafner, J. Ab initio molecular dynamics for open-shell transition metals. *Phys. Rev. B* **48**, 13115-13118 (1993).
11. Kresse, G. & Furthmüller, J. Efficiency of ab-initio total energy calculations for metals and semiconductors using a plane-wave basis set. *Comp. Mater. Sci.* **6**, 15-50 (1996).
12. Hammer, B. *et al.* Improved adsorption energetics within density-functional theory using revised Perdew-Burke-Ernzerhof functionals. *Phys. Rev. B* **59**, 7413-7421 (1999).
13. Gajdos, M. *et al.* CO adsorption on close-packed transition and noble metal surfaces: trends from ab initio calculations. *J. Phys.: Condens. Matter* **16**, 1141–1164 (2004).
14. Schimka, L. *et al.* Accurate surface and adsorption energies from many-body perturbation theory. *Nat. Mater.* **9**, 741-744 (2010).

15. Blöchl, P. E. Projector augmented-wave method. *Phys. Rev. B* **50**, 17953-17979 (1994).
16. Kresse, G. & Joubert, D. From ultrasoft pseudopotentials to the projector augmented-wave method. *Phys. Rev. B* **59**, 1758-1775 (1999).
17. Zhang, F., Li, T., Pan, L., Asthagiri, A. & Weaver, J. F. CO oxidation on single and multilayer Pd oxides on Pd(111): Mechanistic insights from rairs. *Catal. Sci. Technol.* **4**, 3826-3834 (2014).
18. Klikovits, J. *et al.* Surface oxides on Pd(111): STM and density functional calculations. *Phys. Rev. B* **76**, 045405 (2007).
19. Hirvi, J. T., Kinnunen, T. J. J., Suvanto, M., Pakkanen, T. A. & Nørskov, J. K. CO oxidation on PdO surfaces. *J. Chem. Phys.* **133**, 084704 (2010).
20. Nørskov, J. K. *et al.* Origin of the overpotential for oxygen reduction at a fuel-cell cathode. *J. Phys. Chem. B* **108**, 17886-17892 (2004).
21. Dong, A. *et al.* A generalized ligand-exchange strategy enabling sequential surface functionalization of colloidal nanocrystals. *J. Am. Chem. Soc.* **133**, 998-1006 (2011).
22. Fu, Q. *et al.* Interface-confined ferrous centers for catalytic oxidation. *Science* **328**, 1141-1144 (2010).
23. Fernández-García, M. *et al.* New $Pd/Ce_xZr_{1-x}O_2/Al_2O_3$ three-way catalysts prepared by microemulsion: part 1. characterization and catalytic behavior for CO oxidation. *Appl. Catal. B-Environ.* **31**, 39-50 (2001).
24. Priebe, J. B. *et al.* Water reduction with visible light: synergy between optical transitions and electron transfer in Au-TiO_2 catalysts visualized by in situ EPR spectroscopy. *Angew. Chem. Int. Ed.* **52**, 11420-11424 (2013).
25. Gabasch, H. *et al.* In situ XPS study of Pd(111) oxidation at elevated pressure, Part 2: Palladium oxidation in the 10^{-1} mbar range. *Surf. Sci.* **600**, 2980-2989 (2006).
26. Permpoon, S., Berthomé, G., Baroux, B., Joud, J. C. & Langlet, M. Natural superhydrophilicity of sol–gel derived SiO_2–TiO_2 composite films. *J. Mater. Sci.* **41**, 7650-7662 (2006).
27. Abild-Pedersen, F. & Andersson, M. P. CO adsorption energies on metals with correction for high coordination adsorption sites – a density functional study. *Surf. Sci.* **601**, 1747-1753 (2007).
28. Park, S., Yang, P., Corredor, P. & Weaver, M. J. Transition metal-coated nanoparticle films: vibrational characterization with surface-enhanced raman scattering. *J. Am. Chem. Soc.* **124**, 2428-2429 (2002).

29. Zhong, J.H. *et al.* Probing the electronic and catalytic properties of a bimetallic surface with 3 nm resolution. *Nat. Nanotechnol.* **12**, 132-136 (2017).
